# Perceived manageability of debt and mental health during the COVID-19 pandemic: A UK population analysis

**DOI:** 10.1371/journal.pone.0274052

**Published:** 2022-09-21

**Authors:** Mark Shevlin, Enya Redican, Philip Hyland, Sarah Butter, Orla McBride, Todd K. Hartman, Jamie Murphy, Frédérique Vallières, Richard P. Bentall

**Affiliations:** 1 Department of Psychology, Ulster University, Londonderry, Northern Ireland, United Kingdom; 2 Department of Psychology, Maynooth University, Maynooth, Ireland; 3 Department of Social Statistics, University of Manchester, Manchester, England; 4 Centre for Global Health, Trinity College Dublin, Dublin, Ireland; 5 Department of Psychology, University of Sheffield, Sheffield, England; Al-Ahliyya Amman University, JORDAN

## Abstract

**Objectives:**

This study examined the association between perceived manageability of debt and risk of depression, anxiety, and mental health help-seeking among a nationally representative sample of adults living in the United Kingdom (UK).

**Methods:**

Data was derived from the COVID-19 Psychological Research Consortium (C19PRC) Study Wave 6 (August/September 2021) which examined the psychological, social, and economic effects of the COVID-19 pandemic on the UK adult population. Bivariate and logistic regression analyses were conducted to determine the association between different levels of perceived debt manageability (i.e., “easily manageable”, “some problems”, “quite serious problems”, “very serious problems”, “cannot manage at all”) and mental health related outcomes.

**Results:**

Almost a quarter of the sample (24%, n = 494) reported debt management problems, and debt manageability associated with higher levels of anxiety, depression, and mental health help-seeking. After adjusting for demographic variables (e.g. income, receipt of benefits), logistic regression analysis demonstrated a dose-response association between increasing levels of debt manageability problems and mental health outcomes. Specifically, adjusted odds ratios for anxiety ranged from 2.28 (‘some problems’) to 11.18 (‘very serious problems’), for depression ranged from 2.80 (‘some problems’) to 16.21 (‘cannot manage at all’), and for mental health help-seeking ranged from 1.69 (‘some problems’) to 3.18 (‘quite serious problems’, ‘very serious problems’).

**Conclusion:**

This study highlights that debt manageability problems represent a robust predictor of depression, anxiety, and mental-health help seeking.

## Introduction

Described as the “*greatest economic shock experienced by the world economy in decades”* [1], the COVID-19 pandemic and the corresponding public health measures enacted to curb the spread of the virus have profoundly affected individual and household financial resources. While early days of the pandemic saw an increase in personal saving rates [2], the pandemic, overall, contributed to widening global wealth inequality; the lowest earners experienced greatest financial strain, job loss and temporary furlough, as well as depletion of savings and incurring debt to cover lost wages [3]. More recently, the phasing out of pandemic fiscal support, and rising inflation are expected to significantly reduce disposable income, savings, and overall standards of living [4, 5]. Consequently, it is likely that many individuals, particularly those less affluent individuals, will take on additional debt to help alleviate financial pressures in the years to come.

It is well-established that debt can have detrimental effects on mental health independent of other socioeconomic indicators such as income, education, and occupation [6–8]. Research indicates that individuals affected by debt are at greater risk of depression, anxiety, suicide, suicidal ideation, substance abuse, and psychotic disorders [8–11], while accumulation of different types of debt has been linked to increased mental health help seeking [12]. However, not all individuals with debt are susceptible to unfavorable mental health outcomes, thus, determining the dimensions of debt that are associated with greater risk of poor mental health is an important research endeavor [13]. A growing body of evidence suggests that subjective components of debt such as worry, stress, and concern about debt may be more influential in predicting mental health than objective indicators such as actual amount of debt accrued [for review see 8]. Perceived manageability of debt represents one subjective dimension of debt linked to poor mental health. For instance, in a study conducted on low-income Northern Irish households [14], the association between financial stress (indicated if an individual reported persistent debt problems, budget problems, or difficulties in managing financially) and mental health was investigated. In this study, the subjective experience of feeling stressed about debt was strongly linked to poor psychological wellbeing, while objective aspects of debt such as size, type, and number of different lenders provided no additional explanatory power. Moreover, the association between perceptions of debt manageability and depression was examined among a representative sample of UK adults from 1999 to 2005 [15]. Results revealed that roughly 1 in 10 respondents reported financial stress; moreover, this group also reported experiencing higher levels of depressive symptoms.

In this study, we examine the associations between perceived manageability of debt and mental health among a representative sample of UK adults during the COVID-19 pandemic. Aspects of mental health that we sought to investigate included depression, anxiety, and mental health help-seeking. Although research has established a link between financial strain during the pandemic and mental health [16, 17], no study (to the best of our knowledge) has examined the relationship between perceived manageability of debt and mental health during this period. Consistent with existing research indicating increased risk of mental health problems among individuals in debt [9, 10, 13, 18], we hypothesized that perceived problems in managing debt would be associated with higher risk of meeting clinical caseness for depression and anxiety, irrespective of sociodemographic variables (such as household income band and receipt of benefits). Although no study, to the best of our knowledge, has examined the association between perceived problems in managing debt and mental health help-seeking, similar to a prior study’s [12] finding that cumulative debt is linked to increased mental health seeking, it was hypothesized that perceived problems in managing debt would be associated with higher likelihood of mental health help-seeking.

## Materials and methods

### Participants

This study used data collected as part of Wave 6 of the COVID-19 Psychological Research Consortium (C19PRC) Study [19], which was established in March 2020 to assess the long-term psychological, social, and economic impact of the COVID-19 pandemic on the UK population [20]. Briefly, at baseline (Wave 1, 23–28 March 2020), 2,025 adults were recruited via the survey company *Qualtrics*, using stratified quota sampling methods to ensure that the sample characteristics of age, gender and household income were representative of the UK adult population [21]. Data for Wave 6 were collected between 6 August– 28 September 2021. During this period *Qualtrics* conducted data collection in two stages: at Phase 1 (6 Aug– 28 Sept), all participants who had previously taken part in the main strand of the C19PRC Study (at baseline or recruited during subsequent waves) were recontacted. At Phase 2 (8–28 Sept) new participants were recruited to match specific characteristics of the adults lost to panel attrition based on age, gender, and household income. This resulted in a recontacted Phase 1 sample of 1,643 (51.8% retention rate) and 415 new participants recruited at Phase 2. The combined final Wave 6 sample (N = 2,058) closely mirrored the characteristics of the baseline sample and was representative of the UK adult population aged 18 years and older, with respect to gender, age, and household income [19]. The Wave 6 data used in the current study is available at: https://osf.io/qv47z/. Sample characteristics are reported in Table 1.

**Table 1:** Demographic characteristics of the sample.

	N	%
**Gender**		
Male	983	47.8%
Female	1069	51.9%
Transgender	4	0.2%
Prefer not to say/Other	2	0.0%
**Age**		
18–24	213	10.3%
25–34	395	19.2%
35–44	380	18.5%
45–54	422	20.5%
55–64	354	17.2%
65+	294	14.3%
**Ethnicity**		
White British/Irish	1805	87.7%
White non-British/Irish	65	3.2%
Indian	42	2.0%
Pakistani	26	1.3%
Chinese	20	1.0%
Other ethnic group	100	4.70%
**Highest Qualification**		
No Qualifications	61	3.0%
O-level / GCSE or similar	412	20.0%
A-level or similar	400	19.4%
Technical qualification	207	10.1%
Undergraduate degree	558	27.1%
Diploma	73	3.5%
Postgraduate degree	322	15.6%
Other qualifications	25	1.2%
**Employment Status**		
Employed full-time	925	44.9%
Employed part-time	281	13.7%
Self-employed full-time	61	3.0%
Self-employed part-time	55	2.7%
Unemployed, looking for work	83	4.0%
Unemployed, family or home	128	6.2%
Unemployed, sick or disability	122	5.9%
Government ’furlough’ scheme	6	0.3%
Retired	342	16.6%
Full-time student	55	2.7%

#### Ethical consideration

Ethical approval for the study was granted by the University of Sheffield (Ethical approval ref no. 033759). The procedures contributing to this work all comply with the ethical standards of the relevant national and institutional committees on human experimentation and with the Helsinki Declaration of 1975, as revised in 2008. For the C19PRC study, informed electronic consent was provided by all participants prior to completing the survey while participants were advised to contact the NHS website directly should they have any concerns about COVID-19 after completing the survey”.

### Measures

#### Demographic variables

Participants were asked to report their age (years) and gender (Male, Female, Transgender, prefer not to say, Other). Education was initially measured using eight categories ultimately, recoded into a binary variable indicting if the participant had attended post-secondary education (No = 0, Yes = 1). Participants were asked to “*Please choose from the following options to indicate your approximate gross (before tax is taken away) household income in 2019*. *Include income from partners and other family members living with you and all kinds of earnings including salaries and benefits”*. Options included one of five categories: “£0–£300 per week (equals about £0–£1290 per month or £0–15,490 per year)”, “£301–£490 per week (equals about £1,291–£2,110 per month or £15,491–£25,340 per year)”, “£491–£740 per week (equals about £2,111–£3,230 per month or £25,341–£38,740 per year)”, “£741–£1,111 per week (equals about £3,231–£4,830 per month or £38,741–£57,930 per year)”, and “£1,112 or more per week (equals about £4,831 or more per month or £57,931 or more per year)”. Participants were also asked “Are you currently in receipt of any government benefits (not including child benefits and state pension)?” and given the response options Yes (0) and No (1).

#### Manageability of debt

Participants were asked *“There are differences in how manageable people think their debt is*. *How manageable is your level of debt*?*”* with five response options ranging from 1, ‘*My debt is easily manageable*’ to 5 ‘*I cannot manage my debt at all*’. Participants were not asked this question if they indicated in a previous question that they did not have any debt (i.e., *“Has your overall debt increased or decreased this month due to COVID-19*?*”* and answered *“I do not have any debt”*). A manageability of debt variable was then created which included six categories, the lowest being “I do not have any debt.”

#### Major Depressive Disorder (MDD)

The PHQ-9 [22] measures the nine symptoms of MDD described in the fourth edition of the Diagnostic and Statistical Manual of Mental Disorders [23]. Participants are asked to indicate how often they have been bothered by each symptom over the last two weeks on a four-point Likert scale ranging from 0 (*Not at all*) to 3 (*Nearly every day*). Possible scores range from 0–27 with higher scores reflecting higher symptomatology. The recommended and commonly used cut-off score of ≥ 10 was used to identify possible cases of DSM-IV MDD. This cut-off score has been shown to have adequate sensitivity (.85) and specificity (.89) for detecting cases of MDD [22]. The psychometric properties of the PHQ-9 scores have been widely supported [24], hence why the PHQ-9 was selected to measure PHQ-9 in the C19PRC study. The internal reliability of the scale scores in this study was *α* = .94.

#### Generalized Anxiety Disorder (GAD)

The GAD-7 [25] measures seven symptoms of GAD described in the fourth edition of the Diagnostic and Statistical Manual of Mental Disorders [23]. It asks participants to indicate how often they have been bothered by the various symptoms over the last two weeks on a four-point Likert scale that ranges from 0 (*Not at all*) to 3 (*Nearly every day*). Possible scores range from 0–21, where higher scores reflect greater symptomatology. The recommended cut-off score of ≥ 10 was used to identify possible cases of GAD as this cut-off score has been shown to have adequate sensitivity (.89) and specificity (.82) for detecting cases of GAD [25]. The psychometric properties of the GAD-7 scores have been widely supported [26], hence why the GAD-7 was employed as a measure of anxiety in the present study. The internal reliability of the scale scores in this study was *α* = .96.

#### Mental health treatment seeking

Participants were provided with the following information: *“Mental health difficulties are very common*. *It will help us understand our survey results if you would tell us whether you currently or have in the past received treatment (medication or talking therapies) for these kinds of difficulties*.*”* Options were provided, of which the participants were required to tick all that apply: “(1) I have never received treatment for mental health problems, (2) I have received treatment for mental health problems in the past, (3) I am currently receiving treatment for mental health problems, (4) I am currently receiving treatment for mental health problems but it has been cancelled temporarily due to the lockdown, (5) I am currently on a waiting list to receive treatment for a mental health problem, and (6) Prefer not to answer”. A binary variable was created to represent current mental health help seeking by collapsing options 3 and 4 into a single category (Yes = 1; No = 0).

### Data analysis

First, binary variables representing caseness were calculated from the PHQ-9 and GAD-7 total scores, and along with the mental health help-seeking variable, were stratified by the debt manageability variable. Second, the debt manageability variable was dummy-coded with the ‘no debt’ as the reference category. These dummy coded variables were used as predictors in logistic regression models with the binary PHQ-9, GAD-7, and mental health help-seeking variables as dependent variables. The estimates were reported as odds-ratios (OR) and 95% confidence intervals, and these indicate the predicted change in the odds of caseness for the PHQ-9, GAD-7, or mental health help-seeking for each level of the debt manageability variable compared to the ‘no debt’ category. These were the unadjusted estimates. These models were estimated again with age, gender, education, income, and being in receipt of benefits as covariates. The adjusted ORs (AOR) from these models indicate the effects while controlling for these variables.

## Results

The majority of the sample (76.0%; n = 1564) reported having no debt or debt that was ‘easily manageable’ (Table 2). Of the remaining participants, 16.3% (n = 335) reported having “some problems”, 4.4% (n = 90) reported having “quite serious problems”, 2.6% (n = 54) reported having “serious problems”, and 0.7% (n = 15) reported “cannot manage at all”. Higher rates of anxiety, depression, and current mental health treatment were evident among reporting more serious problems in managing their debt (i.e., “quite serious problems,” “very serious problems”, “cannot manage at all”). A reviewer asked about the association between self-reported COVID-19 infection and perceived debt manageability. The association between these variables are reported in S1 Table.

**Table 2:** Bivariate and multivariate binary logistic regression results predicting depression, anxiety and mental health help seeking.

Debt		Anxiety			Depression			Mental Health Help Seeking		
	N	N (%)	OR	aOR	N (%)	OR	aOR	N (%)	OR	aOR
Does not have debt	724 (35.2%)	87 (12.0%)	*	*	107 (14.8%)	*	*	46 (6.4%)	*	*
My debt is easily manageable	840 (40.8%)	123 (14.6%)	1.26 (.93–1.68)	1.13 (.82–1.54)	158 (18.8%)	1.34 (1.02–1.74)	1.26 (.95–1.69)	67 (8.0%)	1.27 (.86–1.88)	1.14 (.76–1.71)
I have some problems managing my debt	335 (16.3%)	114 (34.0%)	3.78 (2.74–5.19)	2.28 (1.62–3.20)	146 (43.6%)	4.45 (3.30–6.00)	2.80 (2.03–3.86)	57 (17.0%)	3.02 (2.00–4.56)	1.69 (1.09–2.61)
I have quite serious problems managing my debt	90 (4.4%)	52 (57.8%)	10.02 (6.23–16.10)	5.18 (3.11–8.61)	61 (67.8%)	12.13 (7.45–19.74)	6.56 (3.89–11.05)	28 (31.1%)	6.65 (3.89–11.38)	3.19 (1.79–5.67)
I have very serious problems managing my debt	54 (2.6%)	41 (75.9%)	23.09 (11.90–44.80)	11.18 (5.55–22.51)	41 (75.9%)	18.19 (9.43–35.07)	8.97 (4.45–18.03)	19 (35.2%)	8.00 (4.24–15.07)	3.18 (1.60–6.33)
I cannot manage my debt at all	15 (.7%)	8 (53.3%)	8.37 (2.96–23.64)	5.23 (1.70–16.08)	12 (80.0%)	23.07 (6.40–83.10)	16.22 (4.17–62.99)	3 (20.0%)	3.68 (1.00–13.51)	1.81 (.46–7.08)

Note: aOR = adjusted odds ratio, adjusted for age (years), gender, education, 2019 household income band, receipt of benefits.

* Reference category for regression analysis

Results from the logistic regression models are reported in Table 2. Compared to those who reported no debt, those who reported any problems managing debt were significantly more likely to meet clinical caseness for anxiety (χ2 (5) = 232.281, p < .001). Inspection of adjusted ORs reveals that, even after adjusting for demographic variables including income bands and receiving benefits, those participants reporting any problems managing debt were significantly more likely to meet clinical caseness for anxiety (χ^2^ (10) = 368.602, p < .001). There was no statistically significant association between debt that was “easily manageable” and anxiety in either model. Compared to the reference group who reported no debt problems, participants reporting any problems managing debt were significantly more likely to meet clinical caseness for depression. This model was statistically significant (χ2 (5) = 232.281, p < .001). Similarly, after adjusting for demographic variables including income bands and receiving benefits, participants reporting any problems managing debt were significantly more likely to meet clinical caseness for depression compared to the reference group. There was evidence of a dose-response association between problems in managing debt and likelihood of meeting caseness for anxiety, with those reporting “cannot manage at all” having the highest likelihood. This adjusted model was statistically significant (χ^2^ (10) = 437.576, p < .001).

The unadjusted logistic regression model including debt manageability and mental health help-seeking was statistically significant (χ^2^ (5) = 87.12, p < .001), with those reporting any problems managing debt being more likely to engage in mental health help-seeking. Similar patterns were observed after adjusting for demographic variables where those participants reporting “some problems”, “quite serious problems”, and “very serious problems” were significantly more likely to engage in mental health help-seeking than those without debt. Surprisingly, there was also no statistically significant association between reporting “cannot manage at all” and mental health help-seeking. This overall model was again statistically significant (χ^2^ (10) = 183.650, p < .001).

## Discussion

This study examined the association between perceived manageability of debt and risk of poor mental health and mental health help-seeking among a representative sample of adults from the UK during the COVID-19 pandemic. The main findings can be summarized succinctly. Firstly, more than three-fourths of the sample (i.e., 76%) reported having either no debt or debt that was “easily manageable”, while a smaller yet substantial quarter of the sample (i.e., 24%) reported problems managing their debt. Second, higher rates of anxiety, depression, and mental health help-seeking were evident among individuals reporting debt problems. Third, a strong and significant association was observed between the various levels of problems in managing debt and risk of anxiety, depression, and mental health help-seeking even after accounting for other potentially influential socioeconomic indicators such as income bands and being in receipt of government benefits. And fourth, there was evidence of a dose-response association between severity of perceived debt manageability and risk of poor mental health. This is important given that dose-response is widely accepted as a factor to consider when judging whether an effect is causal [27].

Consistent with prior research, indicating debt to be a salient risk factor for depression and anxiety [e.g., 9, 10, 13, 18], our findings highlight how clinical depression and anxiety are common among individuals with problems in managing their debt than those not struggling to control their debt. These strong associations remained even after accounting for income and receiving government benefits, supporting prior research indicating debt to be an important risk factor for poor mental health in its own right [e.g., 6, 7, 28]. It was interesting that the likelihood of having clinically significant anxiety symptoms was lower for those reporting “cannot manage at all” compared to “very serious problems”, and that there was no statistically significant association between “cannot manage at all” and mental health help-seeking. This may be attributable to the fewer number of participants in this group reducing power to detect any present effects. Alternatively, the higher prevalence of depression within this subgroup may explain the lower risk of anxiety. Research has shown how shame, guilt, and a sense of personal responsibility are typical emotions experienced by individuals affected by debt [29], and commonly observed features of depression [30]. Therefore, it may be that when an individual perceives their debt as reaching the point of being completely unmanageable that this is likely to be when these feelings of shame, guilt, and personal responsibility are most pervasive. Alternatively, when an individual’s worst-case scenario has already occurred (i.e., debt has become totally uncontrollable), they may be more likely to concede defeat and succumb to their financial reality. Consequently, at this stage, core symptoms of anxiety such as worries about the future [31] may be less relevant than for those reporting “very serious problems” where the threat of more serious debt manageability problems may heighten anxiety. Similarly, the lack of association between uncontrollable debt and mental health help-seeking may indicate that such individuals have better mental health than those individuals where the prospect of more severe debt manageability problems looms. Future research may benefit from investigating factors which explain the relationship between different levels of perceived debt manageability and depression, anxiety, and mental health help-seeking.

Extending previous research investigating the effects of long-term debt on mental health support seeking [12], the findings from the current study illustrate a dose-response association between concerns about debt manageability and mental health support seeking. Determining whether similar associations between debt manageability and mental health help-seeking across other countries is a necessary endeavor. This is especially relevant given that in the UK, the National Health Service (NHS) provides free access to general practitioners, medical prescriptions, and talking therapies, and thus mental health services may be more accessible for individuals affected by debt problems. However, in other countries, such services must be paid for privately. For instance, in the United States, individuals with health insurance are significantly more likely to receive mental health treatment than those without [32]. Given that ability to afford health insurance is largely dependent on an individual’s financial wellbeing, it is likely that those affected by debt are less likely to have the opportunity to engage in mental health help-seeking.

The magnitude of effects of the different levels of debt manageability on mental health outcomes in the current study are substantially greater than prior studies utilizing objective measures such as a study investigating the association between debt and common mental disorders (CMD) where debt was inferred if participants had indicated being in arrears with any utilities, housing-related debts, or shopping-related debts [11]. In this study [11], it was found that after adjusting for demographics, those who reported any debt were over two times more likely to experience a depressive episode (OR = 2.36) and almost three times more likely to experience generalized anxiety disorder (OR = 2.51). Similarly, in a longitudinal investigation of the association between debt and CMD (12), it was found that after adjusting for socio-demographic and socio-economic factors, those with any debt at time point one were almost twice as likely to have a CMD (OR = 1.76), and those reporting three or more debts were almost four times more likely to report a CMD (OR = 3.59). Thus, findings from the current study align with the growing consensus that subjective characteristics of debt often have greater explanatory power than objective characteristics [8, 14, 33, 34]. There are numerous potential explanations for why manageability of debt serves as a powerful predictor of mental health difficulties in the current study. Conservation of resources theory [COR; 35, 36] postulates that psychological stress arises when an individual’s resources, including financial, are threatened, exhausted, or unable to be replenished. If an individual perceives their debt as being beyond their financial means, it is likely that this will trigger a perpetuating cycle of debt-related stress where inability to service debts as per the agreed schedule may result in higher interest rates and ultimately, the prosect of debt collection [37]. This may not always be the case for objective measures of debt such as whether an individual is in arrears, which research has shown to increase risk of depression and anxiety only when the cumulative total exceeds £2000 [15]. In addition, the investigation of subjective perceptions of debt may facilitate the acquisition of knowledge not provided by objective measures such as the total amount of debt [14]. Indeed, enquiring about perceived manageability of debt provides important insights into an individual’s sense of control and capacity to cope with their debt, aspects that are difficult to isolate with objective measures. Third, the socioeconomic context of the current study may also explain the magnitude of the effects observed in the current study. Specifically, there is a growing evidence base documenting the pervasive effects of financial concerns stemming from the COVID-19 pandemic on mental health [16, 38–41]. Whether the unique socioeconomic circumstances of the COVID-19 pandemic contributed to problems with debt manageability and consequent risk of poor mental health is a matter which warrants further investigation.

Notable strengths of this study include the use of a large representative survey ensuring strong generalizability of findings, as well as the examination of perceptions of manageability of debt during the COVID-19 pandemic. It also used a directly worded measure of perceived debt manageability, rather than using the number of debts [7] or asking about stress related to debts [34]. However, this study also has some limitations. First, although the C19PRC study is a longitudinal investigation of the psychological, social, and economic impact of the COVID-19 pandemic, the data utilized in the current study was cross-sectional and thus, inferences regarding causality are prohibited. Thus, it is not possible to ascertain whether concerns regarding debt manageability preceded mental health difficulties and mental health help seeking or vice versa. Research evidence pertaining to the relationship between debt and risk of poor mental health has been heterogenous, with some studies finding evidence for the social causation hypothesis (i.e., economic adversity increases risk for poor mental health) and others for the social drift hypothesis (i.e., poor mental health increases susceptibility to economic adversity) [42]. For instance, a recent study [9] found that the risk of debt accumulation is greater among individuals who have previously suffered from mental health difficulties, while another [43] found that existing mental health difficulties at one time-point did not increase risk of debt at subsequent time points. Moreover, research has shown how those with poorer pre-pandemic mental health were more likely to encounter economic disruptions (i.e., usual economic activity, job loss, loss of income, changes to working hours) due to the pandemic [44]. Therefore, it may be that pre-existing mental health conditions prior to the pandemic coupled with the financial implications of the pandemic may have resulted in the substantial effect sizes observed in the association between perceptions of debt manageability on mental health outcomes in the current study. However, again, the cross-sectional analyses of the current study precluded causal inferences regarding the potential influence of the COVID-19 pandemic. Finally, we recognize that COVID-19 could increase debt manageability problems(due to loss of income etc.), but also that manageability of debt could predict the likelihood of contracting COVID-19 given the well-established socioeconomic gradient in COVID-19 [e.g., 45]. As demonstrated in Supplementary Materials, having been infected with COVID-19 was associated with ‘some’ and ‘serious’ problems managing debt. Future research may benefit from exploring the association between COVID-19 infection status and debt manageability problems, particularly with regard to direction of causality.

In conclusion, this study demonstrates perceptions of debt manageability to be a robust risk factor for mental health (i.e., depression, anxiety, and mental health help-seeking), even after accounting for other important socioeconomic indicators such as income and being in receipt of government benefits. Our findings underscore the importance of considering debt as a socioeconomic factor that poses a significant threat to mental wellbeing and emphasizes the necessity of policy interventions aimed at mitigating the harmful effects of debt on mental health, especially as individuals recover from the economic downturn triggered by the COVID-19 pandemic. Co-ordination between health services and debt management agencies is essential in ensuring that those suffering from the mental health effects of debt are best supported [46], while community-based interventions can also be beneficial in some instances in promoting better mental health outcomes for those struggling financially [47]. This study also demonstrates how continuation of economic supports will be helpful in shielding those at most at risk financially from negative outcomes due to the global pandemic. Protection against housing insecurity, allocation of grants to assist those who acquired debt during the pandemic and to support those struggling to pay their daily costs of living, and the investment and restructuring of the social safety nets are some of the key strategies recommended to help those suffering from debt because of the pandemic [48].

## Supporting information

S1 Table Cross tabulation of COVID-19 infection and debt manageability. (DOCX)
