# Supplementary material for: Perceived manageability of debt and mental health during the COVID-19 pandemic: A UK population analysis
Source: PLoS One. 2022 Sep 21;17(9):e0274052. doi: 10.1371/journal.pone.0274052 (PMC9491596; doi:10.1371/journal.pone.0274052)
Supplement: S1 Table — (DOCX) [file pone.0274052.s001.docx]

Chi-square tests of association was conducted (see Supplementary Table 1) to examine the association between the different debt manageability levels and COVID-19 infection status (i.e., infected, not infected, not sure), with the strength of these associations quantified using Cramer’s *V* (≤ 0.2 = weak, 0.2 - 0.6 = moderate, > 0.6 strong). Cells with adjusted standardized residuals >1.96, indicating a statistically significant association, are bolded.

The chi-square test was significant (χ^2^(10) = 85.43, p < .001; Cramer’s *V* = .14) with a weak association. Having been infected with COVID-19 was associated with ‘some’ and ‘serious’ problems managing debt.

| Supplementary Table 1. Cross tabulation of COVID-19 Infection and Debt Manageability. | | | | | | | | | |
| --- | --- | --- | --- | --- | --- | --- | --- | --- | --- |
| Infected COVID-19 | | | How manageable is your level of debt? | | | | | | Total |
|  |  |  | Does not have debt | My debt is easily manageable | I have some problems managing my debt | I have quite serious problems managing my debt | I have very serious problems managing my debt | I cannot manage my debt at all |  |
|  | No | N | **624** | 661 | **252** | 66 | **27** | 9 | 1639 |
|  |  | % | **86.2%** | 78.7% | **75.2%** | 73.3% | **50.0%** | 60.0% | 79.6% |
|  | Yes | N | **56** | 120 | **58** | 13 | **25** | 4 | 276 |
|  |  | % | **7.7%** | 14.3% | **17.3%** | 14.4% | **46.3%** | 26.7% | 13.4% |
|  | I'm not sure | N | 44 | 59 | 25 | **11** | 2 | 2 | 143 |
|  |  | % | 6.1% | 7.0% | 7.5% | **12.2%** | 3.7% | 13.3% | 6.9% |
